# Perceptions of Health Care Use in Germany during the COVID-19 Pandemic

**DOI:** 10.3390/ijerph17249351

**Published:** 2020-12-14

**Authors:** André Hajek, Freia De Bock, Lothar H. Wieler, Philipp Sprengholz, Benedikt Kretzler, Hans-Helmut König

**Affiliations:** 1 Department of Health Economics and Health Services Research, Hamburg Center for Health Economics, University Medical Center Hamburg-Eppendorf, 20246 Hamburg, Germany; b.kretzler.ext@uke.de (B.K.); h.koenig@uke.de (H.-H.K.); 2 Federal Centre of Health Education, 50825 Cologne, Germany; Freia.DeBock@bzga.de; 3 Robert-Koch-Institute, 13353 Berlin, Germany; WielerLH@rki.de; 4 Department of Health Communication, University of Erfurt, 99089 Erfurt, Germany; philipp.sprengholz@uni-erfurt.de

**Keywords:** COVID-19, SARS-CoV-2, access to health care, availability of medical care, postponed treatments, health care use, health services research, health care utilization

## Abstract

This paper examined the determinants of perceived access to health care use during the COVID-19 pandemic in Germany using data from two waves (8 and 16) of the COVID-19 Snapshot Monitoring (COSMO). Descriptive and regression analysis were used. In wave 8, we found that about 60% of the individuals rather disagreed about having had problems accessing medical care. Furthermore, 73% of the individuals rather disagreed to having experienced health deteriorations due to restrictions on the availability of medical care. Moreover, 85% of the individuals were rather optimistic about future access to healthcare services. Overall, slightly better past and future access to healthcare services has been reported in wave 16. Several determinants were identified in regression analysis. In conclusion, data suggest that perceived past and future access to healthcare services during the COVID-19 pandemic is reasonably good.

## 1. Introduction

The COVID-19 pandemic can have consequences for the medical care of the population, e.g., because capacities need to be adapted, or because the risk of transmission of SARS-CoV-2 needs to be reduced. It is important to know how this affects the subjectively experienced access to care, expected access to care, and the subjective state of health of the population.

With regard to previous literature, two studies [1,2] focused on delayed access among children. More precisely, a recent survey among pediatricians in the UK and Ireland showed that about one third (32%) of the pediatricians witnessed delayed presentations (with diabetes mellitus being the most common delayed presentation) [2]. Another recent study [1] based on 12 cases of delayed access to hospital care in Italy showed that half of the children were admitted to an intensive care unit and four died. All parents reported the risk of SARS-CoV-2 infection as a reason for their avoidance behavior [1].

With regard to the adult population, a recent population survey [3] showed that 26% of the Belgian and Dutch respondents experienced cancelled/postponed care and about nearly one third (32%) were concerned about the availability of medication during the first eight weeks of the partial lockdown (further details regarding the term lockdown are presented elsewhere: [4]). However, since there is a lack of corresponding population-based studies in Germany during the COVID-19 pandemic, the aims of this work are as follows: First, to describe self-reported past access to health care ((a) in terms of problems in accessing medical care and (b) in terms of health deteriorations due to restrictions in the availability of medical care) and to identify corresponding factors. Second, to describe perceived future access to medical care and to identify corresponding factors. Thus, we refer to past (experiences) and future (expectations) perceived access to health care.

Due to fear of being infected with COVID-19, we expect that individuals frequently report problems in accessing medical care, often report health deteriorations due to restrictions in the availability of medical care and expect problems in future access to medical care.

Why is this important? This knowledge about these factors may help to avoid underuse (e.g., not visiting the hospital despite critical events such as stroke or heart attack) [5] and to maintain health during the COVID-19 pandemic [6,7]. Moreover, knowledge about the expectations regarding future access to health care may assist in quantifying concerns with regard to the availability of healthcare services in the general adult population in Germany. In other words: It may quantify the level of trust in the performance of the German health care system. This may indirectly quantify the level of trust in government actions during the COVID-19 pandemic.

It should be emphasized that the aim of this study was to explore the proportion of individuals experiencing challenges in health care use during the COVID-19 pandemic, in terms of problems accessing medical care, health deteriorations due to restrictions in the availability of medical care, and perceived future access to medical care rather than to identify (unexplored) determinants of actual health service use prior to the pandemic like various studies have done [8].

Beginning in mid-March (16 March 2020) first nationwide anti-corona measures (e.g., strict regulations of opening hours for bars or restaurants, school closings or day-care center closings) were implemented in Germany (so called partial lockdown). In the following week (22 March 2020) other measures were implemented such as contact restrictions in public or travel bans. These measures were prolonged in the following weeks. Germany started loosening COVID-19 measures on 20 April 2020. For instance, most shops (smaller than 800 square meters) are being allowed to open again. Furthermore, schools gradually started to reopen from 4 May 2020. COVID-19 measures were loosened in the following weeks (e.g., reopening zoos, playgrounds, museums or houses of worship). Contact restrictions were also eased in May. Further restrictions were loosened in June (e.g., for restaurants). Nevertheless, if the rate in a specific region exceeds a certain rate, the restrictions could be implemented again.

Some key characteristics of the German health care system are worth describing. First, the health insurance system in Germany provides comprehensive protection against health care expenses. In general, health insurance is compulsory and about 90% of the inhabitants are insured by social statutory health insurance (SHI) funds, whereas about 10% are insured by private health insurances (PHI). The membership in the SHI is compulsory for most members, particularly for employees below a certain income-threshold. Employees exceeding this threshold and self-employed individuals may opt for PHI. While contributions to SHI depend on the income and do not depend on the health status, the opposite is the case for premium for PHI. Most expenses of out- and inpatient treatment (and pharmaceuticals) are covered by SHI and PHI. Individuals have free access to General Practitioners (GP) and specialists. Moreover, in case of an emergency and via referral by an outpatient physician, hospital care can be used. In general, waiting times for appointments (outpatient physicians) are quite short [9]. Additional details are given elsewhere [10].

## 2. Materials and Methods

### 2.1. Sample

For this study, repeated cross-sectional data were taken from wave 8 and wave 16 of the COVID-19 Snapshot Monitoring (COSMO) beginning in March 2020. More precisely, the monitoring started on 3–4 March 2020. Subsequent waves took place every upcoming week, with wave 8 taking place from 21 to 22 April 2020 and wave 16 from 7 to 8 July 2020. Both, in wave 8 and wave 16, age of individuals included ranged from 18 to 74 years (German speaking and living in Germany). It should be noted that the individuals taking part in wave 8 are different from those taking part in wave 16. A market research company (Respondi, https://www.respondi.com) recruited the individuals from an online-panel. The distribution of age, gender (crossed-quote: age × gender) and federal state (uncrossed) corresponds to the distribution in the German population [11].

### 2.2. Dependent Variables

Three statements served as outcome measures: (1) “I had problems accessing medical care (e.g., because planned treatments have been postponed)” (2) “Due to restrictions in the availability of medical care, my health has deteriorated” (in both cases: 1 = not applicable because there was no need for care; from 2 = strongly disagree to 8 = strongly agree). (3) “Should you need access to medical care in the next 12 months: How high do you estimate the probability of being able to access medical care?” (1 = extremely unlikely; 2 = not likely; 3 = likely; 4 = very likely). The first two outcome measures explicitly refer to the past four weeks. The face validity of our outcome measures were supported by a pretest with n = 15 individuals.

### 2.3. Independent Variables

Based on the theoretical foundation of the Andersen model [12], the independent variables were selected. The Andersen model differentiates between predisposing characteristics like age or sex, enabling resources like access to health care and need factors such as chronic diseases. Thus, several independent variables were included: sex (men; women), age group (four age categories: 18 to 29 years; 30 to 49 years; 50 to 64 years; 65 years and over), living situation (at least two individuals in the same household; living alone), region (East Germany; West Germany), COVID-19 cases/100,000 population (below median; above median) town size (distinguishing between: municipality/small town (1–20,000 inhabitants); medium sized town (20,001–100,000 inhabitants); small city (100,001–500,000 inhabitants); big city (>500,000 inhabitants)), relationship/marriage (no; yes), presence of children under 18 years (no; yes), years of education (up to 9 years/10 years and more (without general qualification for university entrance); 10 years and more (with general qualification for university entrance)), self-employment (no; yes), migration background (no; yes), presence of at least one chronic disease (no; yes). In addition, with regard to COVID-19, the affect (from 1 to 7; higher values correspond to higher affect) and presumed severity of the disease (“How do you assess an infection with the novel corona virus for yourself?” from 1 to 7; higher values correspond to higher severity) were included in the regression model. With regard to affect, items were for example: “For me, the new type of corona virus is...”—“Concerning” (1) to “not concerning” (7), or—“near” (1) to “far away” (7). The affect score consists of seven items. By averaging the items, the score was developed. Cronbach’s alpha was 0.78 (wave 8) and 0.80 (wave 16).

### 2.4. Statistical Analysis

First, we present some descriptive statistics for the three analytical samples (wave 8): In the first analytical sample, 360 individuals were included. These were individuals who reported a need for care when answering the question regarding “problems accessing medical care”. This means that they selected the answer options from 2 (strongly disagree) to 8 (strongly agree) for this item. In other words: We excluded individuals who answered “not applicable because there was no need for care” (answer option: 1) to the question regarding “problems accessing medical care”.

In the second analytical sample, 374 individuals were included. These were individuals who reported a need for care when answering the question regarding “health deterioration (due to restrictions in the availability of medical care)”. This means that they selected answer options from 2 (strongly disagree) to 8 (strongly agree) for this question. In other words: We excluded individuals who answered “not applicable because there was no need for care” (answer option: 1) to the question regarding “health deterioration (due to restrictions in the availability of medical care)”.

In the third analytical sample, *n* = 976 individuals were included. These were individuals who responded to the question regarding perceived future access to medical care. Potential differences in the three analytical samples are shown in Supplementary Table S1.

Moreover, to gain initial insights into the correlates of perceived future access to medical care, sample characteristics are shown stratified by this outcome measure (third analytical sample with *n* = 976 individuals). Furthermore, more details regarding our outcome measures are presented in histograms. Thereafter, multiple linear regressions were performed to examine the determinants of our three outcome measures.

The criterion for statistical significance was set at *p* < 0.05. Stata 16.0 (Stata Corp., College Station, TX, USA) was used to conduct statistical analyses.

### 2.5. Ethics Statement

Informed consent was obtained from all individual participants included in the study. All procedures performed in the COSMO studies involving human participants were in accordance with the ethical standards of the University of Erfurt institutional research committee and with the 1964 Helsinki Declaration and its later amendments or comparable ethical standards.

## 3. Results

### 3.1. Sample Characteristics

In wave 8 (wave 16 in parentheses), among individuals who reported a need for care (referring to the first analytical sample), approximately 30% (wave 16: about 26%) of the individuals rather agreed about having problems accessing medical care (answer options: 6 to 8), whereas 10% (wave 16: about 9%) were quite indifferent (answer option: 5) and the remaining 60% (wave 16: about 65%) rather disagreed about having problems accessing medical care (answer options: 2 to 4). The average score for problems accessing medical care was 4.1, SD: 2.2 (wave 16, average: 3.9, SD: 2.2).

Among individuals who reported a need for care (referring to the second analytical sample), about 15% (wave 16: about 14%) of the individuals rather agreed about having experienced health deteriorations due to restrictions in the availability of medical care (answer options: 6 to 8), whereas approximately 12% (wave 16: about 8%) were quite indifferent (answer option: 5) and the remaining 73% (wave 16: about 78%) rather disagreed about suffering health deteriorations due to restrictions in the availability of medical care (answer options: 2 to 4). The average score for health deteriorations due to restrictions in the availability of medical care was 3.4, SD: 1.7 (wave 16, average: 3.2, SD: 1.7).

In our third analytical sample, perceived future access (in terms of probability of being able to access medical care) in the next 12 months was as follows: While about 15% (wave 16: about 11%) of the individuals expected the probability of being able to access medical care to be extremely unlikely or not likely, the remaining 85% (wave 16: about 89%) were rather optimistic about future access to healthcare services (“likely”/very likely”).

**Figure 1:**
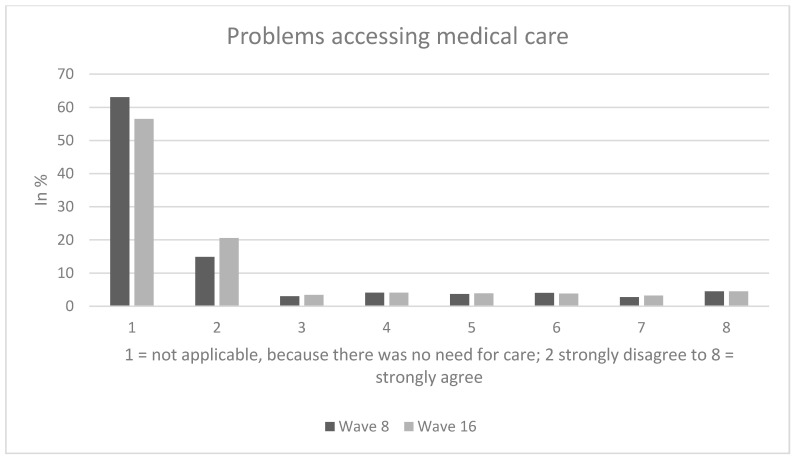
Problems accessing medical care.

**Figure 2:**
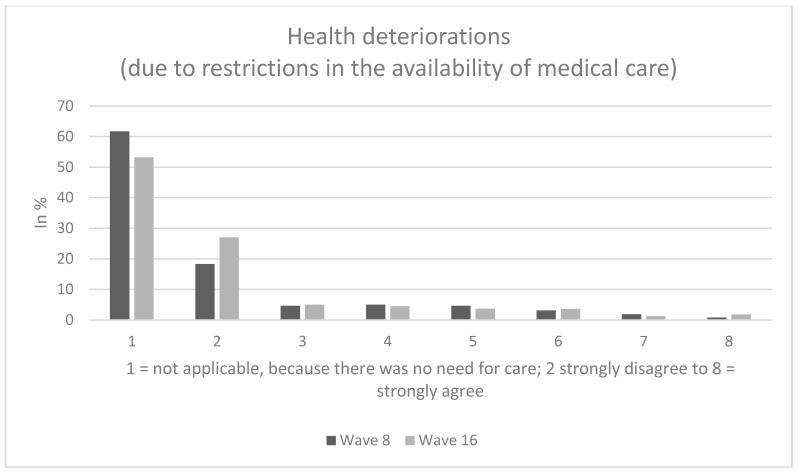
Health deteriorations (due to restrictions in the availability of medical care).

**Figure 3:**
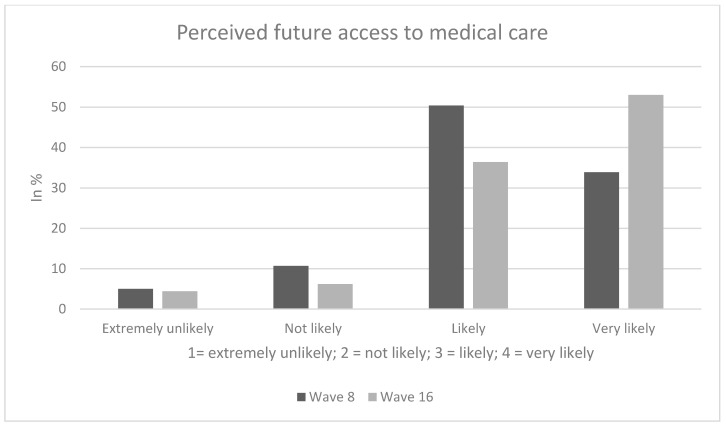
Perceived future access to medical care.

Sample characteristics (wave 8) for the third analytical sample stratified by perceived future access to medical care are shown in Table 1. In our third analytical sample, 48.2% of the individuals were female and average age was 47.0 years (SD: 15.3 years), ranging from 18 to 74 years. Perceived future access was significantly associated with age, presence of children and living situation. Further details are displayed in Table 1. Figure 1, Figure 2 and Figure 3 provide additional details regarding our dependent variables.

It is worth noting that there were no significant differences in the total sample between wave 8 and wave 16 with regard to various sociodemographic factors (e.g., in terms of sex (*p* = 0.86), age (*p* = 0.18), education (*p* = 0.21), region (*p* = 0.43), or migration background (*p* = 0.91)). Some differences exist between wave 8 and wave 16 with regard to two out of our three outcome measures (problems accessing medical care, *p* = 0.11; health deteriorations due to restrictions in the availability of medical care, *p* < 0.05; future access to healthcare services, *p* < 0.001).

### 3.2. Regression Analysis

Multiple linear regressions are shown in Table 2 (wave 8: column 2: with problems accessing medical care as outcome measure; column 3: with health deteriorations due to restrictions in the availability of medical care as outcome measure; column 4; with perceived future access to medical care as outcome measure; findings for wave 16 are displayed in columns 5 to 7). It is worth noting that in the first two regression models, individuals were only included if they reported a need for care.

Regarding wave 8, regressions revealed that better perceptions of access to healthcare services (in terms of (i) fewer problems accessing medical care, (ii) less health deteriorations due to restrictions in the availability of medical care, and (iii) better perceived future access to medical care) was associated with higher age (65 years and over: β = −0.97, *p* < 0.05; β = −0.77, *p* < 0.05; β = 0.30, *p* < 0.05; reference category: 18 to 29 years) and a decreased presumed severity of COVID-19 infection (β = 0.22, *p* < 0.05; β = 0.16, *p* < 0.05; β = −0.07, *p* < 0.001). Moreover, individuals with at least one chronic disease reported better perceived future access to medical care (β = 0.13, *p* < 0.05; compared to individuals without chronic diseases). Individuals with at least one child under 18 years reported more health deteriorations due to restrictions in the availability of medical care (β = 0.63, *p* < 0.01; compared to individuals without children under 18 years).

The majority of associations remained rather similar in wave 16, particularly for the first two outcome measures. However, some discrepancies should be emphasized. The link between presumed severity of COVID-19 infection and all three outcome measures disappeared. While the link between age and perceived future access to medical care vanished, some associations became significant: Perceived better future access was associated with not having children (β = −0.25, *p* < 0.001), being in a partnership/married (β = 0.27, *p* < 0.001), and living alone (β = −0.20, *p* < 0.01).

## 4. Discussion

### 4.1. Aim and Main Findings

Based on repeated cross-sectional data from a nationally representative survey, the purpose of this study was to clarify the proportion of individuals experiencing challenges in health care use during the COVID-19 pandemic, in terms of problems accessing medical care, health deteriorations due to restrictions in the availability of medical care, and perceived future access to medical care. Furthermore, another aim was to identify the correlates of these challenges. Since there is a complete lack of nationally representative studies measuring challenges in health care use during the COVID-19 pandemic, our study markedly extends the current knowledge.

In sum, study findings showed that perceived past and future access to healthcare services during the COVID-19 pandemic is reasonably good—also compared with Belgian and Dutch inhabitants [3]. Furthermore, better perceived access (in terms of fewer problems accessing medical care, less health deteriorations due to restrictions in the availability of medical care, and better perceived future access to medical care) was associated with higher age (65 years and over) and a decreased presumed severity of COVID-19 infection. Additionally, individuals with at least one chronic disease reported better perceived future access to medical care (compared to individuals without chronic diseases). Individuals with at least one child under 18 years reported more health deteriorations due to restrictions in the availability of medical care (compared to individuals without children under 18 years). With some exceptions, similar findings were observed for wave 16.

### 4.2. Previous Research and Possible Explanations

Rather surprisingly, the link between presumed severity of COVID-19 infection and the outcome measures (e.g., perceived future access) disappeared in wave 16. A possible explanation may be that individuals developed a great confidence in the German health care system which may be driven, among other things, by comparisons to other countries who are worse off as well as by individual experiences.

While there was a link between perceived better future access and not having children and living alone, it was also associated with being in a partnership/married in wave 16. We think that this link may be driven by single and couple households (without children). This is supported by the fact that both single households and two-person households reported an average perceived future access of 3.4 (SD: 0.8), whereas three/four-person and five-plus households reported markedly lower perceived access scores (average: 3.3, SD: 0.8 or average 2.9, SD: 1.0, respectively). It may be the case that larger households have more pessimistic views in terms of future views due to time restrictions (e.g., fulfilling family and job obligations) [13].

It should be noted that individuals with chronic diseases reported better perceived future access in wave 8, whereas they reported more problems accessing medical care in wave 16. This may reflect the fact that some individuals did not visit the physician or the hospital despite medical symptoms in the past months (e.g., due to the fear of being infected by COVID-19) [5] which should be examined further [14].

In both wave 8 and wave 16, compared to young individuals aged 18 to 29 years, individuals aged 65 years and over reported less challenges in health care use (in terms of problems accessing medical care, and in terms of health deteriorations due to restrictions in the availability of medical care). However, as stated above, future studies are required, particularly regarding long-term health deteriorations. Furthermore, studies based on qualitative approaches may clarify this link in further detail. For example, a possible explanation may be that individuals aged 18 to 29 years had perceived problems accessing medical care because of perceived prioritization of health care services. Another explanation may be that individuals 18 to 29 years had less time for physician visits (e.g., due to family obligations).

### 4.3. Strengths and Limitations

We would like to highlight some strengths and limitations of this study. To the best of our knowledge, this is the first study quantifying challenges in health care use during the COVID-19 pandemic (in terms of perceived problems accessing medical care, perceived health deteriorations due to restrictions in the availability of medical care, and perceived future access to medical care). A nationally representative online sample was used. This study has a cross-sectional design with inherent limitations. Moreover, this study focused on individuals aged 18 to 74 years. Thus, children and adolescents as well as individuals aged 75 years and over were excluded. A pretest was performed indicating that the dependent variables used in our study showed a quite high face validity. While several explanatory variables were included in our regression model, it should be noted that some factors are missing such as (perceived) access to healthcare [8], type of health insurance (statutory health insurance vs. private health insurance) [15] functional ability/disability [16], lifestyle factors such as body-mass-index [17] or psychosocial/personality-related factors [18,19].

## 5. Conclusions

Data suggest that perceived past access to healthcare services during the COVID-19 pandemic is reasonably good. Encouragingly, individuals in old age reported good perceived access in the past months. Moreover, knowledge about the factors associated with poorer perceived access (e.g., individuals with children) may assist in avoiding underuse of health services in these risk groups (e.g., avoiding health care use despite health needs), and in maintaining health during the COVID-19 pandemic.

With regard to the expectations (perceived future access to healthcare services), the large majority was rather optimistic about future access to healthcare services. This suggests that most individuals are not concerned about future access to healthcare services in Germany but rather have a high level of trust in the performance of the German health care system.

Further studies explicitly focusing on oldest old individuals during this pandemic are urgently required. Additionally, given the international differences in available care facilities per inhabitant and differences of COVID-19-infections requiring intensive care unit (ICU)-care, future studies in other countries (including developing countries [20]) are needed.

## Figures and Tables

**Table 1:** Sample characteristics for the analytical sample stratified by perceived future access to medical care (*n* = 976 individuals) at wave 8.

Independent Variables	Access: Extremely Unlikely (*n* = 49; 5.0%)	Access: Not Likely (*n* = 104; 10.7%)	Access: Likely (*n* = 492; 50.4%)	Access: Very Likely (*n* = 331; 33.9%)	*p*-Value
	**Mean (SD)/*n* (%)**	**Mean (SD)/*n* (%)**	**Mean (SD)/*n* (%)**	**Mean (SD)/*n* (%)**	
Sex					0.38
Men	22 (4.7)	53 (11.3)	225 (47.9)	170 (36.1)	
Women	27 (5.3)	51 (10.1)	267 (52.8)	161 (31.8)	
Age category					<0.001
18 to 29 years	6 (3.8)	16 (10.0)	96 (60.4)	41 (25.8)	
30 to 49 years	27 (7.1)	52 (13.8)	195 (51.6)	104 (27.5)	
50 to 64 years	11 (3.8)	21 (7.3)	144 (49.8)	113 (39.1)	
65 years and over	5 (3.3)	15 (10.0)	57 (38.0)	73 (48.7)	
Children under 18 years:					<0.01
No	30 (4.2)	67 (9.3)	360 (50.3)	259 (36.2)	
Yes	19 (7.3)	37 (14.2)	132 (50.8)	72 (27.7)	
Education					0.26
up to 9 years/10 years and more (without general qualification for university entrance)	19 (4.4)	53 (1.24)	205 (47.9)	151 (35.3)	
10 years and more (with general qualification for university entrance)	30 (5.5)	51 (9.3)	287 (52.4)	180 (32.8)	
Town size					0.69
Municipality/small town (1–20,000)	17 (4.6)	48 (13.0)	180 (48.8)	124 (33.6)	
Medium sized town (20,001–100,000)	13 (5.6)	22 (9.6)	121 (52.6)	74 (32.2)	
Small city (100,001–500,000)	10 (5.7)	15 (8.5)	83 (47.2)	68 (38.6)	
Big city (>500,000)	9 (4.5)	19 (9.5)	108 (53.7)	65 (32.3)	
Region					0.39
West Germany	45 (5.4)	92 (11.0)	414 (49.7)	282 (33.9)	
East Germany	4 (2.8)	12 (8.4)	78 (54.5)	49 (34.3)	
Cases/100,000 population					0.60
Below median	21 (4.7)	51 (11.3)	234 (52.0)	144 (32.0)	
Above median	28 (5.3)	53 (10.1)	258 (49.1)	187 (35.5)	
Relationship/Marriage					0.05
No	13 (4.3)	26 (8.7)	141 (47.0)	120 (40.0)	
Yes	36 (5.3)	78 (11.6)	351 (51.9)	211 (31.2)	
Living situation					<0.01
Living alone	12 (4.9)	18 (7.4)	108 (44.3)	106 (43.4)	
At least 2 individuals in the same household	37 (5.1)	86 (11.7)	384 (52.5)	225 (30.7)	
Migration background					0.17
No	37 (4.4)	89 (10.6)	425 (50.6)	289 (34.4)	
Yes	12 (8.8)	15 (11.0)	67 (49.3)	42 (30.9)	
Self-employment					0.46
No	42 (4.8)	91 (10.3)	449 (51.0)	298 (33.9)	
Yes	7 (7.3)	13 (13.5)	43 (44.8)	33 (34.4)	
Chronic disease					0.16
No	38 (6.0)	71 (11.2)	319 (50.4)	205 (32.4)	
Yes	11 (3.2)	33 (9.6)	173 (50.5)	126 (36.7)	
Affect: COVID-19 infection (from 1 to 7; higher values correspond to higher affect)	4.4 (1.0)	4.4 (1.0)	4.4 (1.0)	4.2 (1.0)	0.40
Severity: COVID-19 infection (from 1 to 7; higher values correspond to higher severity)	4.0 (1.7)	4.2 (1.6)	4.0 (1.5)	3.8 (1.6)	0.05

**Table 2:** Determinants of problems accessing medical care (column 2), health deteriorations (due to restrictions in the availability of medical care; column 3), and perceived future access to medical care (column 4). Results of multiple linear regressions.

	Wave 8			Wave 16		
**Independent Variables**	**Problems Accessing Medical Care**	**Health Deteriorations (due to Restrictions in the Availability of Medical Care)**	**Perceived Future access to Medical Care**	**Problems Accessing Medical Care**	**Health Deteriorations (due to Restrictions in the Availability of Medical Care)**	**Perceived Future access to Medical Care**
Gender: Female (Ref.: Male)	0.27 (0.24)	−0.18 (0.18)	−0.04 (0.05)	0.08 (0.21)	−0.06 (0.17)	−0.09 + (0.05)
Age category: 30 to 49 years (Ref.: 18 to 29 years)	$-$0.09 (0.33)	−0.45 + (0.27)	−0.01 (0.08)	0.0 (0.33)	0.20 (0.25)	0.00 (0.07)
50 to 64 years	0.03 (0.40)	−0.58 + (0.30)	0.22 ** (0.08)	−0.33 (0.37)	−0.38 (0.27)	0.08 (0.08)
65 years and over	−0.97 * (0.43)	−0.77 * (0.35)	0.30 ** (0.09)	−0.90 * (0.39)	−0.62 * (0.27)	0.08 (0.10)
Children (under 18 years): Yes (Ref.: Absence of children under 18 years)	0.25 (0.27)	0.63 ** (0.23)	−0.06 (0.07)	0.10 (0.30)	0.47 * (0.24)	−0.25 *** (0.07)
Education: General qualification for university entrance (Ref.: absence of qualification for university entrance)	−0.22 (0.26)	−0.26 (0.19)	0.07 (0.05)	−0.02 (0.24)	−0.22 (0.18)	0.08 (0.05)
Town size: - Medium sized town (20,001–100,000) (Ref.: municipality/small town (1–20,000))	−0.16 (0.31)	−0.24 (0.23)	0.01 (0.07)	−0.16 (0.28)	−0.20 (0.20)	0.05 (0.06)
Small city (100,001–500,000)	0.29 (0.35)	0.06 (0.26)	0.06 (0.07)	−0.63 + (0.32)	−0.12 (0.25)	−0.02 (0.08)
Big city (>500,000)	−0.49 (0.32)	−0.28 (0.25)	0.05 (0.07)	−0.04 (0.30)	0.34 (0.24)	−0.08 (0.08)
Region: East Germany (Ref.: West Germany)	−0.22 (0.36)	−0.04 (0.26)	0.09 (0.08)	0.17 (0.32)	0.13 (0.25)	−0.01 (0.07)
Cases/100,000 population: Above median (Ref.: below median)	0.13 (0.26)	0.20 (0.20)	0.07 (0.06)	0.06 (0.24)	0.01 (0.18)	0.04 (0.06)
Relationship/Marriage: Yes (Ref.: no partnership/marriage)	0.45 (0.36)	0.40 (0.25)	−0.04 (0.07)	−0.22 (0.29)	−0.34 (0.23)	0.27 *** (0.07)
Living situation: At least 2 individuals in the same household (Ref.: living alone)	−0.54 (0.41)	−0.36 (0.27)	−0.07 (0.07)	0.57 + (0.34)	0.45 + (0.26)	−0.20 ** (0.08)
Migration background: Yes (Ref.: no migration background)	−0.35 (0.29)	0.35 (0.25)	−0.12 (0.08)	−0.02 (0.30)	0.30 (0.23)	−0.06 (0.08)
Self-employment: Yes (Ref.: not self-employed)	−0.25 (0.37)	−0.17 (0.27)	−0.10 (0.09)	−0.06 (0.36)	0.32 (0.30)	0.04 (0.08)
Chronic disease: Yes (Ref.: no chronic diseases)	−0.03 (0.26)	−0.22 (0.20)	0.13 * (0.06)	0.48 * (0.24)	0.25 (0.18)	0.05 (0.06)
Affect: COVID-19 infection (higher values correspond to higher affect)	−0.27 + (0.14)	−0.08 (0.10)	−0.00 (0.03)	−0.18 (0.12)	−0.11 (0.09)	0.02 (0.03)
Severity: COVID-19 infection (higher values correspond to higher severity)	0.22 * (0.09)	0.16 * (0.07)	−0.07 *** (0.02)	0.12 (0.08)	0.08 (0.06)	0.02 (0.02)
Constant	5.16 *** (0.93)	4.02 *** (0.68)	3.15 *** (0.20)	3.68 *** (0.84)	3.26 *** (0.62)	3.14 *** (0.19)
Observations	360	374	976	425	458	978
R^2^	0.07	0.09	0.06	0.06	0.10	0.06

Unstandardized beta-coefficients are reported; robust standard errors in parentheses; *** *p* < 0.001, ** *p* < 0.01, * *p* < 0.05, + *p* < 0.10. The outcome measures were assessed as follows (first two outcome measures from 2 = strongly disagree to 8 = strongly agree): “I had problems accessing medical care (e.g., because planned treatments have been postponed)”; “Due to restrictions in the availability of medical care, my health has deteriorated.”; “Should you need access to medical care in the next 12 months: How high do you estimate the probability of being able to access medical care?” (1 = extremely unlikely; 2 = not likely; 3 = likely; 4 = very likely).

## Supplementary Materials

The following are available online at https://www.mdpi.com/1660-4601/17/24/9351/s1, Table S1: Sample characteristics for the three analytical samples (wave 8).
