# Supplementary material for: Perceptions of Health Care Use in Germany during the COVID-19 Pandemic
Source: Int J Environ Res Public Health. 2020 Dec 14;17(24):9351. doi: 10.3390/ijerph17249351 (PMC7764965; doi:10.3390/ijerph17249351)
Supplement: Supplementary file 1 [file ijerph-17-09351-s001.pdf]

Table S1. Sample characteristics for the three analytical samples (wave 8).

Variables	First analytical sample (*n* = 360)	Second analytical sample (*n* = 374)	Third analytical sample (*n* = 976)
Sex			
Men	183 (50.8%)	187 (50.0%)	470 (48.2%)
Women	177 (49.2%)	187 (50.0%)	506 (51.8%)
Age category			
18 to 29 years	50 (13.9%)	50 (13.4%)	159 (16.3%)
30 to 49 years	148 (41.1%)	156 (41.7%)	378 (38.7%)
50 to 64 years	105 (29.2%)	111 (29.7%)	289 (29.6%)
65 years and over	57 (15.8%)	57 (15.2%)	150 (15.4%)
Children under 18 years:			
No	243 (67.5%)	260 (69.5%)	716 (73.4%)
Yes	117 (32.5%)	114 (30.5%)	260 (26.6%)
Education			
up to 9 years / 10 years and more (without general qualification for university entrance)	161 (44.7%)	169 (45.2%)	428 (43.9%)
10 years and more (with general qualification for university entrance)	199 (55.3%)	205 (54.8%)	548 (56.1%)
Town size			
Municipality/small town (1–20.000)	136 (37.8%)	141 (37.7%)	369 (37.8%)
Medium sized town (20,001–100,000)	94 (26.1%)	101 (27.0%)	230 (23.6%)
Small city (100,001–500,000)	59 (16.4%)	62 (16.6%)	176 (18.0%)
Big city (> 500,000)	71 (19.7%)	70 (18.7%)	201 (20.6%)
Region			
West Germany	301 (83.6%)	311 (83.2%)	833 (85.3%)
East Germany	59 (16.4%)	63 (16.8%)	143 (14.7%)
Cases/100,000 population			
Below median	172 (47.8%)	183 (48.9%)	450 (46.1%)
Above median	188 (52.2%)	191 (51.1%)	526 (53.9%)
Relationship/Marriage			
No	95 (26.4%)	111 (29.7%)	300 (30.7%)
Yes	265 (73.6%)	263 (70.3%)	676 (69.3%)
Living situation			
Living alone	75 (20.8%)	87 (23.3%)	244 (25.0%)
At least 2 individuals in the same household	285 (79.2%)	287 (76.7%)	732 (75.0%)
Migration background			
No	299 (83.1%)	315 (84.2%)	840 (86.1%)
Yes	61 (16.9%)	59 (15.8%)	136 (13.9%)
Self-employment			
No	318 (88.3%)	332 (88.8%)	880 (90.2%)
Yes	42 (11.7%)	42 (11.2%)	96 (9.8%)
Chronic disease			
No	198 (55.0%)	196 (52.4%)	633 (64.9%)
Yes	162 (45.0%)	178 (47.6%)	343 (35.1%)
Affect: COVID-19 infection (from 1 to 7; higher values correspond to higher affect)	4.3 (1.0)	4.4 (1.0)	4.3 (1.0)
Severity: COVID-19 infection (from 1 to 7; higher values correspond to higher severity)	4.2 (1.6)	4.3 (1.6)	4.0 (1.5)
